# Perseverative Cognition as an Explanatory Mechanism in the Relation Between Job Demands and Sleep Quality

**DOI:** 10.1007/s12529-017-9683-y

**Published:** 2017-09-12

**Authors:** Michelle Van Laethem, Debby G. J. Beckers, Sabine A. E. Geurts, Johanna Garefelt, Linda L. Magnusson Hanson, Constanze Leineweber

**Affiliations:** 1 Behavioural Science Institute, Radboud University, Nijmegen, The Netherlands; 2 Department of Work and Organizational Psychology, University of Amsterdam, Amsterdam, The Netherlands; 3 Stress Research Institute, Stockholm University, Stockholm, Sweden

**Keywords:** Bidirectional, Job stressors, Reciprocal relations, Rumination, Work demands, Work preoccupation

## Abstract

**Purpose:**

The aim of this longitudinal three-wave study was to examine (i) reciprocal associations among job demands, work-related perseverative cognition (PC), and sleep quality; (ii) PC as a mediator in-between job demands and sleep quality; and (iii) continuous high job demands in relation to sleep quality and work-related PC over time.

**Method:**

A representative sample of the Swedish working population was approached in 2010, 2012, and 2014, and 2316 respondents were included in this longitudinal full-panel survey study. Structural equation modelling was performed to analyse the temporal relations between job demands, work-related PC, and sleep quality. Additionally, a subsample (*N* = 1149) consisting of individuals who reported the same level of exposure to job demands during all three waves (i.e. stable high, stable moderate, or stable low job demands) was examined in relation to PC and sleep quality over time.

**Results:**

Analyses showed that job demands, PC, and poor sleep quality were positively and reciprocally related. Work-related PC mediated the normal and reversed, direct across-wave relations between job demands and sleep quality. Individuals with continuous high job demands reported significantly lower sleep quality and higher work-related PC, compared to individuals with continuous moderate/low job demands.

**Conclusion:**

This study substantiated reciprocal relations between job demands, work-related PC, and sleep quality and supported work-related PC as an underlying mechanism of the reciprocal job demands-sleep relationship. Moreover, this study showed that chronically high job demands are a risk factor for low sleep quality.

**Electronic supplementary material:**

The online version of this article (doi:10.1007/s12529-017-9683-y) contains supplementary material, which is available to authorized users.

## Introduction

Sleep problems are prevailing in modern society, with about one third of individuals from Western countries suffering from poor sleep [1, 2]. Suboptimal sleep quality is associated with negative health consequences and deficient work performance [3–5] and is characterized by one or more of the following symptoms [6]: (i) difficulties initiating sleep, (ii) difficulties maintaining sleep, (iii) waking up too early, or (iv) feeling non-refreshed in the morning.

Previous research has shown that (chronic) stress is an essential antecedent of poor sleep quality and that work can be an important cause of stress [7–9]. Work-related stress can be defined as emotional, cognitive, behavioural, and physiological reactions to negative aspects of work [10]. A recent review suggests that job demands are among the most important work-related stressors in relation to sleep complaints [11, 12]. Moreover, several recent studies based on the Swedish Longitudinal Occupational Survey of Health (SLOSH) cohort found that job demands are adversely and longitudinally related to two important dimensions of poor sleep quality: sleep disturbances and non-restorative sleep (i.e. awakening problems) [13, 14] (Garefelt et al., submitted). However, not all studies have provided consistent support for these findings [15]. Knowledge about the temporal job demands-sleep relation is still limited, and little is known about possible underlying mechanisms of this relationship [12].

Work-related perseverative cognition (PC) may play an important role in the pathway from job demands to reduced sleep quality [16, 17]. PC is defined as “repeated or chronic activation of the cognitive representation of one or more psychological stressors” [17] (p. 114), with work-related PC resulting from work-related issues [18]. Specifically, the PC hypothesis suggests that a continuous mental representation of (work) stressors may cause prolonged physiological activation and, consequently, poor stress recovery and poor sleep, rather than (or in addition to) the stressors themselves [17]. Especially work-related PC is assumed to jeopardize psycho-physiological recovery from job demands and has accordingly been associated with work-related stress(ors) and reduced sleep quality [19–21].

### Reciprocal Relations Between Job Demands, Work-Related PC, and Sleep Quality

Only few studies have examined the direction of temporal relations between job demands, work-related PC, and sleep quality. Studies that did focus on these interrelations found indications for reciprocal relations between these concepts [13, 20]. Thus, in addition to normal causation relations (job demands → PC, PC → sleep quality), also reversed causation relations (sleep quality → PC, PC → job demands) were detected. This reversed causal path is explained by the ‘stressor creation hypothesis’ [22–24], which states that poor sleep quality may alter an individual’s perception of their work environment and/or may foster work-related PC.

The first aim of this study was to confirm the reciprocal temporal associations between job demands, work-related PC, and sleep quality. The present study is a follow-up study of (Garefelt et al., submitted) and thus, we expected to replicate the reciprocal, positive relations (i.e. both normal and reversed relations) found between job demands and poor sleep quality (both sleep disturbances and awakening problems; hypothesis 1). Moreover, based on a previous study by Van Laethem et al. [20], we expected that job demands are reciprocally and positively related to work-related PC (hypothesis 2) and that work-related PC, in turn, is reciprocally and positively related to poor sleep quality (hypothesis 3). The second aim of this study was to examine whether perseverative cognition mediates the assumed reciprocal association between job demands and sleep quality. Very few studies on this topic included three or more waves allowing for proper mediation analysis, and no study previously examined reciprocal mediation. We expected that work-related PC is a mediator in the reciprocal temporal association between job demands and sleep quality (hypothesis 4).

### Continuous Exposure to High Job Demands

The core assumption of effort-recovery theory is that after effort expenditure at work, individuals are fully recovered once psycho-physiological systems have returned to baseline levels by means of psycho-physiological unwinding before the start of a new period of effort expenditure [25]. However, if psycho-physiological recovery is incomplete (e.g. due to excessive overwork or prolonged mental preoccupation with work), one starts the next working period still feeling fatigued. As a result, one has to expend compensatory effort to perform adequately, which increases the burden on the recovery process and may lead to an accumulation of fatigue. Accordingly, allostatic load theory states that due to a long-term accumulation of load effects, psycho-physiological systems may start to malfunction, resulting in chronic load effects [26]. This is also in line with the accumulation model [27, 28], which describes the adverse temporal relationship between stressor and strain. The accumulation model posits that this temporal relationship is the result of an accumulation of stress effects, which do not necessarily disappear when the stressor is removed. Based on the allostatic load theory and the accumulation model and as sleep is undeniably the most important recovery activity [7], continuous/chronic high job demands are expected to be associated with a decline in sleep quality. Additionally, work-related PC will likely increase when dealing with higher job demands [7]. The third aim of the present longitudinal study was to examine whether individuals suffering from *continuous* (i.e. long-term) high job demands experience a deterioration in sleep quality and an increase in work-related PC over time. We hypothesized that individuals, who experience stable high job demands, experience lower sleep quality (hypothesis 5a) and higher work-related PC (hypothesis 5b) compared to individuals with stable moderate or low job demands. We also expected that employees with stable high job demands show a decrease in sleep quality (hypothesis 6a) and an increase in work-related PC (hypothesis 6b) over time, whereas these unfavourable changes are expected to be absent among workers with stable low or moderate exposure to job demands.

To examine the reciprocal relations between job demands, work-related PC, and sleep quality, as well as the continuous exposure to job demands, we chose to perform a longitudinal study with three measurement waves each two years apart. A two-year time lag has been identified as an appropriate time lag when assessing stressors in relation to mental health [29, 30] and has frequently been used in high-quality research on job demands and sleep quality [15, 31]. In addition, when focusing on long-term associations, it is preferable to employ a time lag which is rather long as opposed to a short time lag as a longer time lag may merely lead to an underestimation of the actual causal effect, whereas a too short time lag may wrongfully indicate that there is no causal effect [28].

## Methods

### Study Population

The study population consisted of the participants of the SLOSH study, a longitudinal cohort survey with a focus on the association between work organization, work environment, and health. SLOSH follows participants of the Swedish Work Environment Surveys (SWES) conducted every second year by Statistics Sweden. The SWES consist of a subsample of gainfully employed people aged 16–64 from the Labour Force Survey (LFS). Since the start of SLOSH in 2006, eligible SWES participants were invited every second year to respond to a postal questionnaire in two versions, one for those currently gainfully employed and one for those permanently or temporarily employed outside the labour force. Respondents were categorized as being in paid work if they had worked on average ≥ 30% during the past 3 months. Data collection was conducted by Statistics Sweden. The current paper included participants who were gainfully employed in the 2010 (*N* = 9132), 2012 (*N* = 7325), and 2014 (*N* = 15,359) data collections. Out of the 9132 participants employed in 2010, 754 participants were not employed 2 years later, and 2818 people did not respond to the questionnaire in 2012, resulting in 5569 participants gainfully employed in both 2010 and 2012, which corresponds to a response rate of 61%. Out of those, 662 participants were not in paid employment in 2014 and 1765 persons did not participate in SLOSH 2014 at all. Thus, 4079 participants were gainfully employed in all three waves and the response rate from wave 2 to wave 3 was 73%. Dropout analyses were conducted, comparing the effective longitudinal sample with participants with available data at 2010, but who did not participate in the later waves. The analyses showed that the effective longitudinal sample consisted of slightly more women (57.6 vs. 54.3%, *p* < 0.01), somewhat younger (49.2 ± 8.7 vs. 50.2 ± 11.2, *p* < 0.001), and higher educated individuals (3.3 ± 1.4 vs. 2.9 ± 1.4, *p* < 0.001) compared to all respondents in 2010. As the present study focused on sleep quality, respondents who worked night shifts on at least one of the waves were excluded from analyses (*N* = 999). The final sample consisted of 3080 participants. The SLOSH study has been approved by the Regional Research Ethics Board in Stockholm. Informed consent was obtained from all individual participants included in the study.

### Measures

We assessed two important dimensions of sleep quality. *Sleep disturbances* (reflecting a lack of sleep continuity) were measured with four items (difficulty falling asleep, repeated awakenings, early awakening, and disturbed sleep). *Awakening problems* (reflecting feelings of being insufficiently restored) were assessed by two items (difficulty awakening and not well-rested). All items were derived from the Karolinska Sleep Questionnaire (KSQ) [13, 32, 33]. Response options reached from 1 = never to 6 = always/five times a week or more. Cronbach’s alpha coefficients for disturbed sleep ranged from 0.84 to 0.85.

*Job demands* were measured by the Swedish version of the Demand-Control Questionnaire (DCQ) [34–36] and were assessed by four items (working fast, too much effort, enough time (reversed), and conflicting demands). All items had four response options (1 = never/almost never, 2 = rarely, 3 = sometimes, and 4 = often). Cronbach’s alpha coefficients for job demands ranged from 0.65 to 0.68. *Decision authority* was derived from the same questionnaire, was assessed with two questions (choice in how you do your work and what you do at work; the correlation coefficient between the two items ranged from 0.60 to 0.62), and was used as a covariate in this study.

*Work-related PC* was measured with three items from the over-commitment scale from the Effort-Reward Imbalance Questionnaire [37, 38]. These items fit well with the definition of work-related PC presented in the “Introduction”, which posits that one is not able to cognitively detach from work stressors while being at home [18]. The items are as follows: “Work rarely lets me go, I even think about it in the evenings”; “When I get home, I can easily relax and ‘switch off’ work” (reversed); and “As soon as I get up in the morning I start thinking about work problems”. All items were answered on a four-point Likert scale reaching from 1 = totally disagree to 4 = totally agree. Cronbach’s alpha coefficient was 0.83 across all time points.

### Analytic Strategy

Average scores for sleep disturbances, awakening problems, job demands, work-related PC, and the covariate decision authority were computed. The longitudinal data were analysed with structural equation modelling. This type of analysis allows not only for path analysis in the traditional direction (i.e. normal causation: job demands → PC → sleep quality) but also for paths opposite to the traditional direction (i.e. reversed causation: sleep quality → PC → job demands) [39]. The sleep quality dimensions ‘sleep disturbances’ and ‘awakening problems’ were entered as separate, but correlated factors. We compared four possible models: the first model (model 0) only included auto-regressions over time. In the next step, the *normal causation* model (model 1: job demands → sleep quality, job demands → PC, PC → sleep quality) and the *reversed causation* model (model 2: sleep quality → job demands, PC → job demands, sleep quality → PC) were fitted to the data, while still including the auto-regressions in each model. Finally, the reciprocal model (model 3: job demands ↔ sleep quality, job demands ↔ PC, PC ↔ sleep quality) was tested. Structural equation modelling was performed with the lavaan 5.20 package in R Statistical computing and graphics software [40, 41]. To reduce possible bias by missing data, we used the full information maximum likelihood (FIML) estimation [42]. Based on the recommendations of Hu and Bentler [43], model fit was assessed with the root mean square error of approximation (RMSEA) and the comparative fit index (CFI). Standardized estimates were calculated for all models and are reported here. Previous research has shown that several variables are related to job demands, work-related PC, and sleep and may distort results if not accounted for (cf. [20]). For instance, sleep problems are more common in women and increase with age [44, 45]. Thus, the analysis was controlled for sex (1 = male, 2 = female), age (in years), education (1 = compulsory, 2 = two-year upper secondary/vocational training, 3 = 3- or 4-year upper secondary, 4 = university or equivalent < 3 years, 5 = university or equivalent ≥ 3 years), and the time-varying covariates shift work (0 = no shift work, 1 = shift work; night workers were already excluded) and decision authority. Indirect effects (mediated effects) were estimated by the product of coefficients method [46]. In this method, the estimate of the relation between the independent variable and the mediator is multiplied with the estimate of the relation between the mediator and the dependent variable. Statistical significance of the effect was evaluated using the bootstrapped 95% confidence intervals with 10,000 iterations. Mediation is determined when the confidence interval does not contain 0 [47].

For the analysis concerning continuous exposure to high job demands, three subgroups were created. For each time point, mean scores on job demands were divided with a tertiary split. The tertiary split was identical on all time points. The job demands measure was recoded so that 1 indicated low job demands (cut-off score ≤ 2.25), 2 indicated moderate job demands (cut-off score 2.25–2.75), and 3 indicated high job demands (cut-off score > 2.75). If an individual’s score fell into the same demands subgroup on all time points, this individual was categorized into a stable group (i.e. 1-1-1 = stable low job demands group, 2-2-2 = stable moderate job demands group, and 3-3-3 = stable high job demands group). After creating the stable job demands groups, 488 participants (15.8% of full sample) had stable low job demands at all three time points, 277 participants (9.0% of full sample) experienced stable moderate job demands, and 384 participants (12.5% of full sample) had stable high job demands. Altogether, the stable groups (*N* = 1149) comprised of 37.3% of the total sample. Continuous high job demands were high in absolute terms as the cut-off for high job demands was a score between 2.8 and 4 (range 1–4) on all time points. Next, a 3 × 3 repeated measures MANOVA was performed, including time as within factor (i.e. three time points), and sleep disturbances, awakening problems, and work-related PC as dependent variables. Job demands group (stable low, stable moderate, stable high) was entered as between-subject factor, and all covariates were accounted for.

## Results

### Descriptive Statistics

The study sample consisted of slightly more female respondents than male respondents (59% female). Most respondents were ≥ 40 years of age (*M*_baseline_ = 49.01; range 23–71 years). Moreover, the majority of respondents were moderately to highly educated and did not participate in shift work. See Table 1 for characteristics of the full sample as well as the subsample that was used for the group analyses regarding continuous exposure to job demands. Comparing the subgroup to the full sample in terms of sex, age, educational level, and work schedule, no differences were detected (i.e. *d* < 0.10). Lastly, occupation of participants was classified according to the Swedish Standard Classification of Occupations. Distribution of occupations was diverse and represents the Swedish working population well. The most common occupation categories were professionals (e.g. engineers, doctors, teachers; 24.9%), technicians and associate professionals (e.g. computer assistants, photographers, air traffic controllers; 28.3%), and service workers and shop sales workers (e.g. cooks, childcare workers, hairdressers; 13.3%).

**Table 1:** Sample characteristics

	Final full-panel sample		Subsample continuous job demands	
	*N*	%	*N*	%
Sex				
Men	1259	40.9	447	38.9
Women	1821	59.1	702	61.1
Age				
23–29	48	1.6	13	1.1
30–39	430	14.0	149	13.0
40–49	999	32.4	366	31.9
50–59	1299	42.2	498	43.3
60–71	304	9.9	123	10.7
Educational level				
Compulsory	311	10.1	124	10.8
2-year upper secondary/vocational training	724	23.5	267	23.2
3- or 4-year upper secondary	714	23.2	263	22.9
University or equivalent < 3 years	451	14.6	169	14.7
University or equivalent ≥ 3 years	880	28.6	326	28.4
Work schedule (at T1)				
No shift work	2817	91.5	1052	91.6
Shift work	263	8.5	97	8.4
Occupation (at T1)				
Legislators, senior officials, and managers	230	7.6	82	7.2
Professionals	756	24.9	277	24.4
Technicians and associate professionals	860	28.3	320	28.2
Clerks	269	8.9	98	8.6
Service workers and shop sales workers	405	13.3	166	14.6
Skilled agricultural and fishery workers	26	0.9	13	1.2
Craft and related trades workers	246	8.1	93	8.2
Plant and machine operators and assemblers	158	5.2	58	5.1
Elementary occupations	89	2.9	28	2.5

Means, standard deviations, and correlations are presented in Table S1 in the supplemental material. All correlations between the main research variables were significant and in the expected direction. Stability of variables over time was high with standardized beta coefficients ranging between 0.53 and 0.69.

### Interrelations Between Job Demands, Work-Related PC, and Sleep Quality

**Fig. 1:**
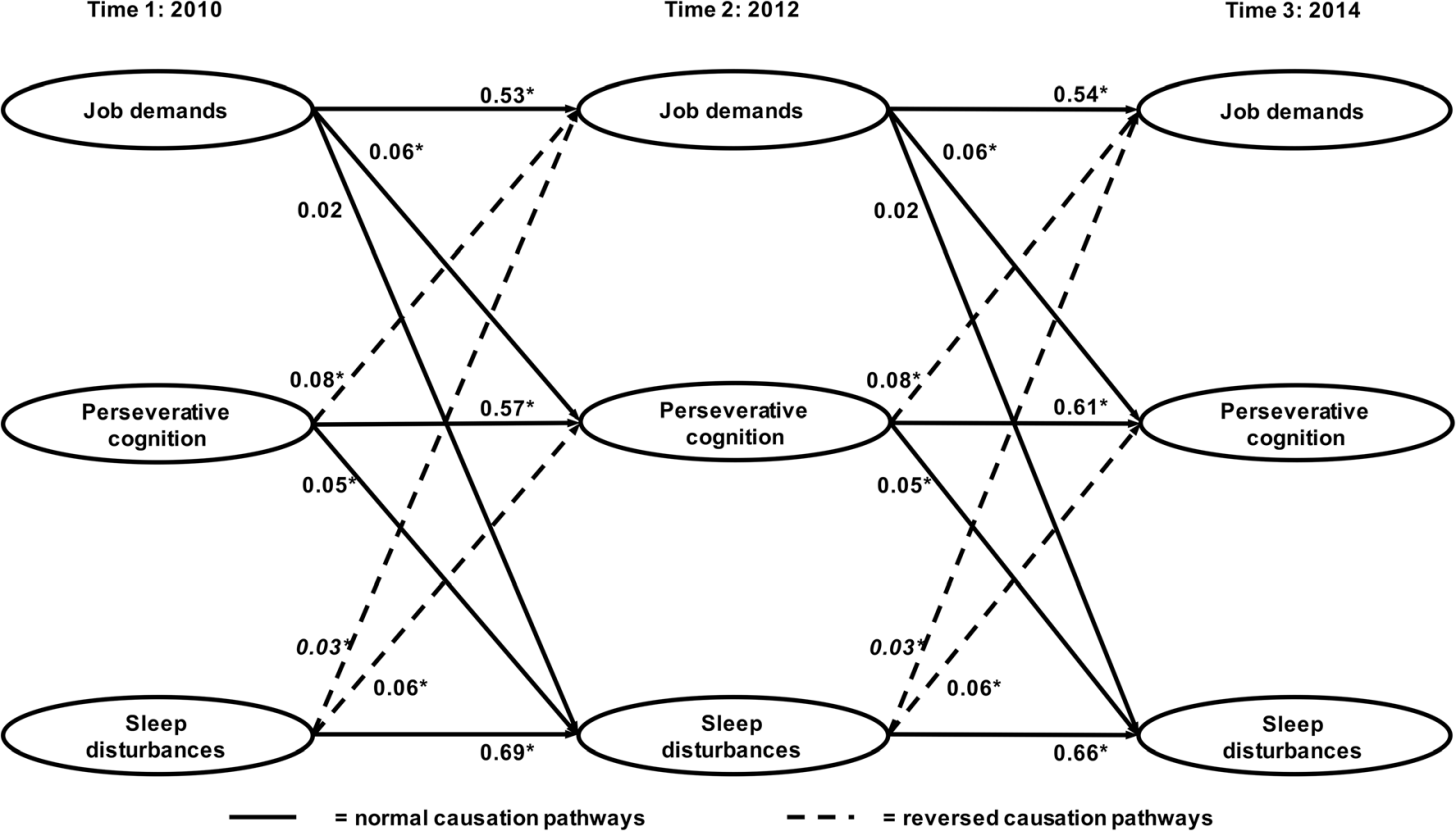
Overview of the normal and reversed pathways (sleep disturbances) and standardized regression coefficients (*β*). The model is adjusted for age, sex, educational level, work schedule, and decision authority, but for clarity, these pathways are not depicted. *p < 0.05

**Fig. 2:**
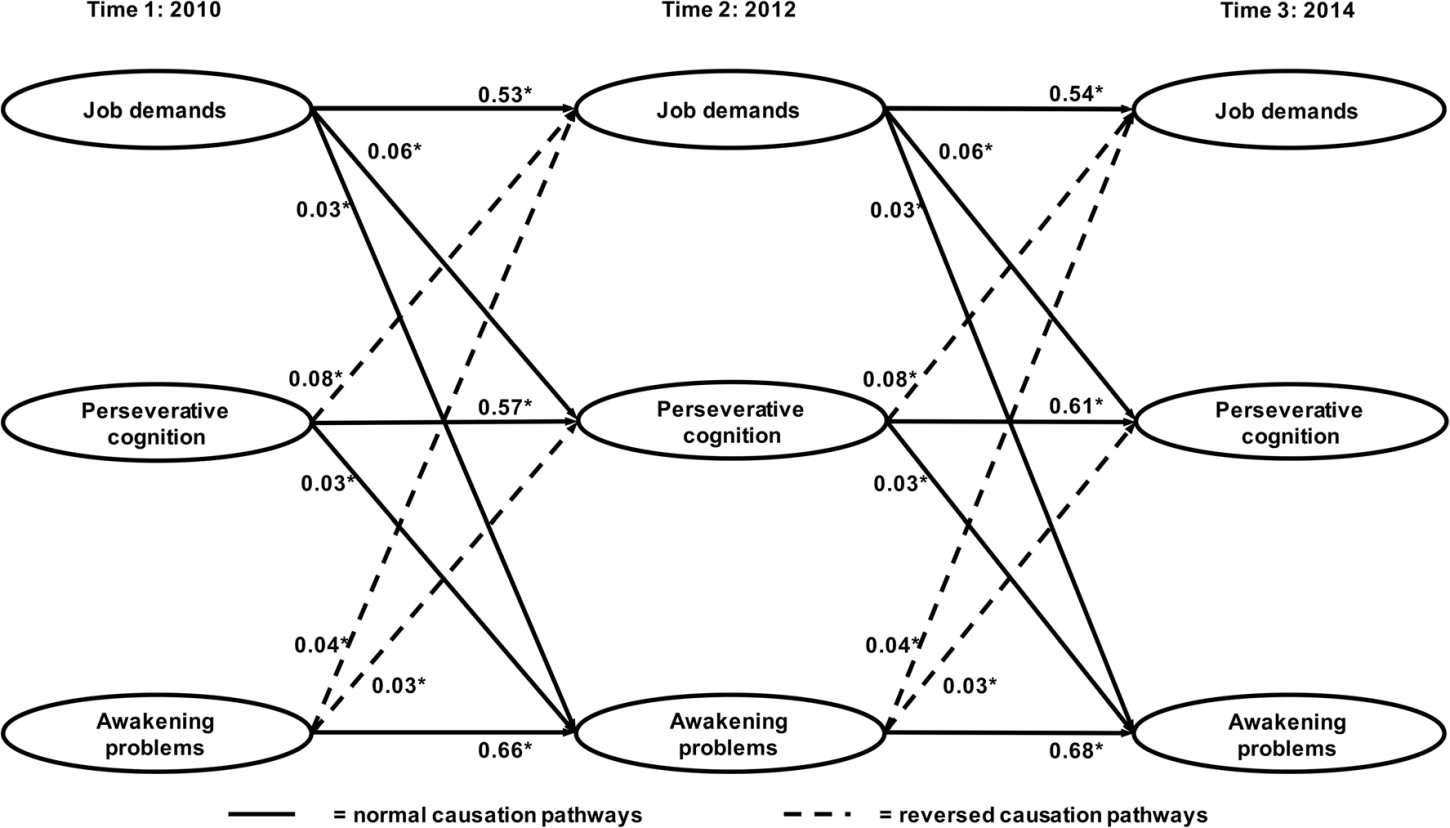
Overview of the normal and reversed pathways (awakening problems) and standardized regression coefficients (*β*). The model is adjusted for age, sex, educational level, work schedule, and decision authority, but for clarity, these pathways are not depicted. *p < 0.05

Structural equation models with constrained normal and reversed pathways (i.e. pathways from T1 to T2 and from T2 to T3 were ‘forced’ to be equal) did not fit the data worse than models including free pathways. Thus, only the constrained models are reported here. All structural equation models fitted the data reasonably well. See Table 2 for an overview of model fit and model comparisons of all structural equation models. We performed chi-square difference tests to compare the normal (model 1), reversed (model 2), and reciprocal (model 3) models to the null model (model 0). We found that all of these models fitted the data significantly better than the null model, which indeed implies a temporal relationship between job demands, work-related PC, and sleep quality. The reciprocal model was shown to fit the data most accurately, providing the strongest support for reciprocal relations between job demands, work-related PC, and sleep quality. See Figs. 1 and 2 for the standardized regression coefficients of the forward and reversed structural paths.

**Table 2:** Model fit and comparisons for structural equation models

Model	Model fit			Model comparison			
	*χ* ^2^ (*df*)	RMSEA	CFI	Model	*χ* ^2^	Model	*χ* ^2^
Model 0	1750.43 (112)	0.069	0.923				
Model 1 (normal)	1653.98 (105)	0.069	0.927	1 vs. 0	96.44*		
Model 2 (reversed)	1588.89 (105)	0.068	0.930	2 vs. 0	161.53*		
Model 3 (reciprocal)	1513.69 (98)	0.068	0.933	3 vs. 0	236.73*	3 vs. 2	75.20*

*p < 0.05

Our results regarding the direct, across-wave (T1–T2, T2–T3) relationship between job demands on the one hand, and sleep disturbances and awakening problems on the other hand, while not yet including work-related PC, replicated the reciprocal, positive relations found by (Garefelt et al., submitted) (see Figure S1 in the supplemental material for a visual representation of the relationships and standardized regression coefficients). As soon as work-related PC was included in the model, however, the across-wave (T1–T2, T2–T3), reciprocal relations between job demands and disturbed sleep disappeared and only near-significant, reversed relations from disturbed sleep to job demands prevailed. The positive, reciprocal relations between job demands and awakening problems remained unchanged. All normal and reversed relations between job demands and both sleep quality dimensions from T1 to T3 (i.e. across four years) were insignificant.

Relations of job demands with work-related PC were positive and reciprocal, which indicates that job demands are not only related to subsequent work-related PC but work-related PC is also related to a subsequently higher experience of job demands. The normal causation and reversed causation pathways were all revealed to be significant. Work-related PC, in turn, was positively and reciprocally related to sleep disturbances and awakening problems as all normal and reversed causation pathways were significant. See Fig. 1 for an overview of relationships concerning sleep disturbances and Fig. 2 for all relationships concerning awakening problems. Please note that all relations concerning sleep disturbances and awakening problems were entered in the same model, but for clarity, are shown in two separate figures.

Since all main effects (i.e. direct, across-wave relations between job demands and both sleep quality dimensions, relations between job demands and work-related PC, and relations between work-related PC and both sleep quality dimensions) were reciprocal, mediation through work-related PC seemed plausible. As requirements for modern mediation were fulfilled (i.e. proof of mediation does not require the independent and dependent variables to be directly related; it suffices to have significant associations between independent variable and mediator as well as between mediator and dependent variable; cf. [48, 49]), we performed a mediation analysis to test whether work-related PC acted as a mediator in between job demands on the one hand and sleep disturbances and awakening problems on the other hand. Specifically, we examined whether work-related PC at T2 mediated the relation between job demands at T1 and sleep disturbances and awakening problems at T3. Since all relations were reciprocal, we also tested reversed mediation effects, in which sleep disturbances and awakening problems at T1 affect job demands at T3 via work-related PC at T2. The confidence interval of the indirect effect from job demands to sleep disturbances via work-related PC did not contain 0. Therefore, work-related PC did act as a mediator in the normal pathway between job demands and sleep disturbances and accounted for approximately 17% of this relation (i.e. the proportion mediated). The proportion mediated is an effect size of the mediation effect and is calculated by dividing the indirect effect by the total effect [50]. Work-related PC was also a mediator in the reversed pathway from sleep disturbances to job demands and mediated about 63% of this association. Lastly, work-related PC was a mediator in the normal and reversed relations between job demands and awakening problems. Work-related PC mediated approximately 13% of the normal pathway from job demands to awakening problems and fully mediated the reversed pathway from awakening problems to job demands. None of the total effects (i.e. the sum of the direct and indirect effect) were significant, which may be due to the insignificant direct effects (T1–T3) between job demands and both sleep quality dimensions. See Table 3 for an overview of all indirect (i.e. the amount of mediation) and total effects as well as standardized estimates.

**Table 3:** Indirect effect (amount of mediation) and total effect (sum of indirect and direct effects) of job demands on sleep disturbances and awakening problems via work-related PC and the reversed effects

Direction of effect	Type of effect	Standardized estimate	Unstandardized estimate (CI)
Job demands (T1) → PC (T2) → sleep disturbances (T3)	Indirect	0.003*	0.005* (0.003–0.008)
Job demands → sleep disturbances	Total	0.018	0.034 (− 0.023 to 0.092)
Sleep disturbances (T1) → PC (T2) → job demands (T3)	Indirect	0.005*	0.003* (0.001–0.004)
Sleep disturbances → job demands	Total	− 0.008	− 0.004 (− 0.025 to 0.016)
Job demands (T1) → PC (T2) → awakening problems (T3)	Indirect	0.002*	0.004* (0.001–0.007)
Job demands → awakening problems	Total	0.016	0.031 (− 0.029 to 0.093)
Awakening problems (T1) → PC (T2) → job demands (T3)	Indirect	0.002*	0.004* (0.001–0.007)
Awakening problems → job demands	Total	− 0.002	0.002 (− 0.019 to 0.023)

*CI* confidence interval

*p < 0.05

### Continuous Exposure to High Job Demands

We performed a repeated measures MANOVA to examine whether individuals exposed to continuous high job demands experience a deterioration in sleep quality and an increase in work-related PC over time. No significant multivariate interaction effect of group and time was revealed (*F*(12, 6606) = 1.67, *p* = 0.07, *η*_p_^2^ = 0.003). Neither was the multivariate within-subjects main effect significant, indicating that the dependent variables (i.e. sleep disturbances, awakening problems, work-related PC) did not change over time (*F*(6, 4402) = 1.46, *p* = 0.18, *η*_p_^2^ = 0.002). However, the between-subjects main effect was significant, showing that the stable demands groups significantly and consistently differed from each other (*F*(6, 2200) = 73.74, *p* < 0.001, *η*_p_^2^ = 0.17).

**Fig. 3:**
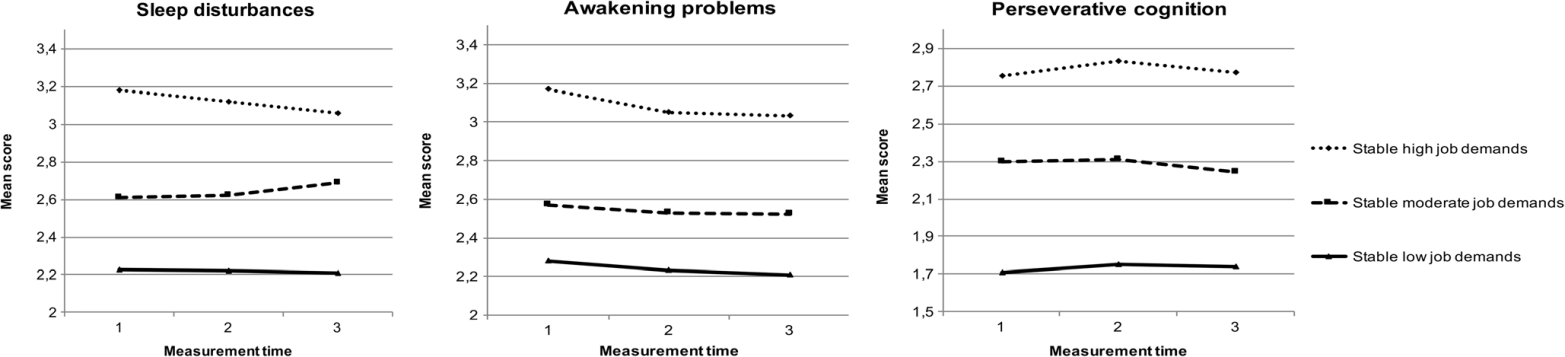
Overview of group effects for stable low, stable moderate, and stable high job demands on sleep disturbances, awakening problems, and work-related PC

Univariate analyses of between-subjects effects revealed that the stable low, stable moderate, and stable high job demands groups differed from each other for disturbed sleep, awakening problems, and work-related PC [disturbed sleep: *F*(2, 1101) = 75.72, *p* < 0.001, *η*_p_^2^ = 0.12; awakening problems: *F*(2, 1101) = 56.49, *p* < 0.001, *η*_p_^2^ = 0.09; work-related PC: *F*(2, 1101) = 263.47, *p* < 0.001, *η*_p_^2^ = 0.32]. Pairwise comparisons with Bonferroni correction supported these findings. Respondents in the stable high job demands group reported highest scores on sleep disturbances, awakening problems, and work-related PC. Respondents in the stable moderate and stable low job demands group reported moderate and lowest scores on sleep disturbances, awakening problems, and work-related PC, respectively. The three job demands groups all differed significantly on sleep disturbances, awakening problems, and work-related PC on all time points. An overview of group effects is presented in Fig. 3.

## Discussion

### Job Demands, Work-Related PC, and Sleep Quality

The first goal of this longitudinal three-wave study was to examine the interrelations between job demands, work-related PC, and sleep quality over time. Results revealed reciprocal, direct across-wave relations between job demands on the one hand and sleep disturbances and awakening problems on the other hand. However, no relations were found between job demands and both sleep quality dimensions across the four-year time span. These findings correspond with previous research, mostly based on the same cohort, which also found reciprocal relations [13, ] and largely supports hypothesis 1. It is noteworthy that the normal prospective relation from job demands to sleep disturbances disappeared when including work-related PC in the measurement model. Previous research has suggested that work-related PC may mediate the stress-sleep relationship [20], which may explain the slightly different findings when including work-related PC in the model.

Job demands and work-related PC were positively and reciprocally related over time. Thus, job demands were related to work-related PC two years later, and work-related PC was related to subsequent job demands. Additionally, positive and reciprocal prospective relations between work-related PC and both sleep quality dimensions (i.e. sleep disturbances and awakening problems) were revealed. Work-related PC was related to future sleep disturbances and awakening problems, which were, in turn, related to subsequent work-related PC. The results regarding positive, reciprocal relations between job demands and work-related PC on the one hand, and work-related PC and sleep quality on the other hand, are in line with a previous study [20] and support hypotheses 2 and 3. Moreover, the findings underline the relevance of examining work-related PC as a promising mediator in the temporal association between job demands and sleep quality.

The mediation analyses indeed showed that work-related PC may act as a mediator in between job demands and sleep quality (i.e. sleep disturbances and awakening problems), which is in line with the PC hypothesis [16, 17] and supports hypothesis 4. The PC hypothesis states that a continuous mental representation of stressors may cause prolonged physiological activation and, consequently, poor sleep quality, rather than (or in addition to) the stressors themselves. Job demands at T1 were associated with an increase in work-related PC at T2, which, in turn, was related to an increase in sleep disturbances and awakening problems at T3. However, the reverse was also true: sleep disturbances and awakening problems at T1 were associated with increased work-related PC at T2, which, in turn, was related to increased job demands at T3. Interestingly, the reversed mediation pathways were stronger compared to the normal mediation pathways. The finding that work-related PC served as a mediator in the reversed relation between sleep quality and job demands extends the PC hypothesis and supports the stressor creation hypothesis [22–24], which suggests that poor sleep may lead to an increase in (perceived) stressors. Indeed, poor sleep may contribute to daytime sleepiness and low performance, which, in turn, may elicit work-related PC. Work-related PC may then increase *actual* job demands, for instance, because one needs to redo certain tasks. Alternatively, one may have a gloomier perspective on work or may lose time due to PC and may *perceive* the same demands as being higher.

### Continuous Exposure to Job Demands

To study continuous exposure to job demands over time, we examined a subgroup of the initial sample with stable job demands across all time points, i.e. stable high, stable moderate, or stable low job demands for (at least) four years. The second objective of this study was to examine whether continuous high job demands lead to decreased sleep quality and increased work-related PC. In line with our hypotheses 5a and 5b and with the effort-recovery theory [25], results showed that individuals with continuous high job demands had lower sleep quality (i.e. more sleep disturbances and awakening problems) and higher work-related PC, compared to individuals with continuous moderate and low job demands. However, contrary to our hypotheses 6a and 6b and not in line with the allostatic load theory [26] and the accumulation model [27, 28], sleep quality did not decrease and work-related PC did not increase over time for individuals experiencing stable high job demands. This finding suggests that most respondents with continuous high job demands experienced already high job demands before entering the study and the accumulating effects of long-term job demands may have reached their maximum. Possibly, after several years of high job demands, negative effects may cease to increase and remain constant.

### Strengths, Limitations, and Suggestions for Future Research

The present study has several assets. First, this study has a longitudinal full-panel design, which allows for drawing, albeit cautious, conclusions about temporal precedence of variables. Moreover, the three-wave longitudinal design permitted us to perform proper mediation analyses. Another strength is the attention given to reciprocal relations instead of only focusing on the traditional direction of causality (i.e. normal causation). A final asset is the investigation of work-related PC as a crucial underlying mechanism of the reciprocal stressor-sleep relationship.

Nonetheless, the present study has some limitations, which need to be taken into account when interpreting the results. A first issue is the exclusive use of self-report measures to assess job demands, work-related PC, and sleep quality. The use of self-report measures has been associated with several problems such as social desirability or retrospection [51]. Spector [52], however, has argued that these issues may not be as problematic as previously believed, for instance because mono-method correlations among study variables are often not higher than multi-method correlations. Related to the validity of the PC measure, one could note the use of three items from the over-commitment scale from the Effort-Reward Imbalance Questionnaire [37, 38] to measure work-related PC. Comparing the definition of work-related PC with the content of the items leads to the conclusion that there is sufficient content validity.

A second limitation is the conservative approach to inclusion of participants. For example, participants only taking part in the first two waves were not included in this study. One might argue that this conservative approach to participant inclusion leads to an underestimation of associations due to low power. However, our sample set was sufficiently powered (i.e. achieved power of 0.95 to detect small effects) and the non-response analyses demonstrate that participants who dropped out only marginally differed from the final study sample. Thus, systematic differences between the participants and dropouts are unlikely and lead us to believe that our conservative approach to data inclusion is warranted. In addition, given the diversity in participants’ occupations, our sample represents the Swedish workforce well and may also be indicative of other European working populations. However, careful conclusions are necessary as more research is needed to expand generalizability.

Another limitation of this study is the time lag of two years between waves, which appears to be rather long for assessing work-related PC. No consensus exists regarding an optimal time lag when measuring the association between sleep and work-related factors. Therefore, future studies may perform studies with varying time lags (e.g. from short time lags of 1 day to longer time lags of two or three years). Especially regarding PC, studies with shorter (day-to-day or week-to-week) time lags are relevant, as apart from stable trait levels of PC, within-person variance (state levels of PC) is plausible and interesting to examine [53]. Please note that, given the time lag of two years used in the present study, we cannot be certain whether the stable job demands groups were actually continuously exposed to stable demands during the entire two years between measurements, or whether job demands fluctuated somewhat in between, but still resulted in generally high job demands. Yet, the high auto-correlations strongly suggest that the stable groups were indeed exposed to overall stable job demands. As is often the case in longitudinal research, effect sizes were rather small (*β* values ranging from 0.03 to 0.08). However, small effect sizes do not imply small effects in relative terms. When establishing changes over time in structural equation modelling, baseline levels of the variables under study are controlled for and explain a large part of the variance [54]. Moreover, job demands and work-related PC are only a few of many factors that have an impact on sleep quality (see [28]). Other important causes of poor sleep quality are, for example, health, stressors in private life, and alcohol use [55, 56]. Consequently, although our study reports small effect sizes in absolute terms, these effects should not be underestimated.

A final limitation is that even though this study sheds more light on causality of relations, longitudinal field studies cannot definitively unravel all causal processes. Consequently, other research designs, as for instance experimental designs, are often proposed to provide more insight into causality. Experimental designs in highly controlled environments are, however, are hard to achieve when addressing the associations among *real-life* variables like job demands, work-related PC, and sleep quality.

### Practical Implications

Knowledge of underlying mechanisms of the stressor-sleep relationship may benefit employers and employees alike. Sleep problems and other health issues stemming from continuous high job demands and a preoccupation with work may be prevented by providing employees with sufficient time to recover from high work load and consequently decreasing work-related PC, e.g. by means of sufficient work breaks during the workday or more control over work schedules (aiding recovery opportunities after work). A recent longitudinal study, for example, showed that lunch breaks are important for recovery during the working day and are related to energy levels at work [57]. Sufficient recovery opportunities and limiting exposure to very high job stressors can prevent a vicious cycle among work stressors, PC, and sleep problems, in the long term aiding both employee health and performance. Also, offering relaxation training could be a way to decrease employees’ PC, by giving employees the means to deal with or prevent adverse repetitive thoughts about work [58]. Another possible method to reduce PC is mindfulness meditation, which has been shown to reduce perseverative modes of thinking [59–61]. Finally, as we found reciprocal effects in this study, it is important to acknowledge that sleep quality may be a valuable point of attack. For instance, promoting good sleep hygiene may help in preventing sleep problems and thus also reduce work-related PC and job demands.

To conclude, the present longitudinal study provided more insight into reciprocity of relations between job demands, work-related PC, and sleep quality. Additionally, the role of work-related PC as an underlying mechanism of the stressor-sleep relationship was strengthened. Lastly, this study provided evidence that individuals with continuous high job demands experience lower sleep quality and higher work-related PC compared to employees with low to moderate job demands. Our findings give a reason to continue focusing on PC in future research on work stress(ors) and sleep.

## Electronic supplementary material

Table S1 (DOCX 32 kb)

Figure S1 Overview of the normal and reversed pathways and standardized regression coefficients (β). The model is adjusted for age, sex, educational level, work schedule, and decision authority, but for clarity these pathways are not depicted. * = *p* < 0.05 (GIF 42 kb)

High resolution image (TIFF 66782 kb)

## References

[1] LeBlanc M, Mérette C, Savard J, Ivers H, Baillargeon L, Morin CM (2009). Incidence and risk factors of insomnia in a population-based sample. Sleep.

[2] Ohayon MM, Reynolds CF (2009). Epidemiological and clinical relevance of insomnia diagnosis algorithms according to the DSM-IV and the International Classification of Sleep Disorders (ICSD). Sleep Med.

[3] Cappuccio FP, D’Elia L, Strazzullo P, Miller MA (2010). Sleep duration and all-cause mortality: a systematic review and meta-analysis of prospective studies. Sleep.

[4] Swanson LM, Arnedt JT, Rosekind MR, Belenky G, Balkin TJ, Drake C (2011). Sleep disorders and work performance: findings from the 2008 National Sleep Foundation Sleep in America poll. J Sleep Res.

[5] Taylor DJ, Lichstein KL, Durrence HH, Reidel BW, Bush AJ (2005). Epidemiology of insomnia, depression, and anxiety. Sleep.

[6] Edinger JD, Bonnet MH, Bootzin RR, Doghramji K, Dorsey CM, Espie CA (2004). Derivation of research diagnostic criteria for insomnia: report of an American Academy of Sleep Medicine Work Group. Sleep.

[7] Åkerstedt T, Nilsson PM, Kecklund G, Sonnetag S, Perrewé PL, Ganster DC (2009). Sleep and recovery. Research in occupational stress and well-being: current perspectives on job-stress recovery.

[8] Crawford ER, LePine JA, Rich BL (2010). Linking job demands and resources to employee engagement and burnout: a theoretical extension and meta-analytic test. J Appl Psychol.

[9] Häusser JA, Mojzisch A, Niesel M, Schulz-Hardt S (2010). Ten years on: a review of recent research on the job demand–control (-support) model and psychological well-being. Work Stress.

[10] Levi L, Levi I (2000). Guidance on work-related stress. Spice of life, or kiss of death?.

[11] Van Laethem M, Beckers DGJ, Kompier MAJ, Dijksterhuis A, Geurts SAE (2013). Psychosocial work characteristics and sleep quality: a systematic review of longitudinal and intervention research. Scand J Work Environ Health.

[12] Linton SJ, Kecklund G, Franklin KA, Leissner LC, Sivertsen B, Lindberg E (2015). The effect of the work environment on future sleep disturbances: a systematic review. Sleep Med Rev.

[13] Åkerstedt T, Garefelt J, Richter A, Westerlund H, Magnusson Hanson LL, Sverke M (2015). Work and sleep—a prospective study of psychosocial work factors, physical work factors, and work scheduling. Sleep.

[14] Magnusson Hanson LL, Chungkham HS, Åkerstedt T, Westerlund H (2014). The role of sleep disturbances in the longitudinal relationship between psychosocial working conditions, measured by work demands and support, and depression. Sleep.

[15] Magnusson Hanson LL, Åkerstedt T, Naswall K, Leineweber C, Theorell T, Westerlund H (2011). Cross-lagged relationships between workplace demands, control, support, and sleep problems. Sleep.

[16] Brosschot JF (2010). Markers of chronic stress: prolonged physiological activation and (un)conscious perseverative cognition. Neurosci Biobehav Rev.

[17] Brosschot JF, Gerin W, Thayer JF (2006). The perseverative cognition hypothesis: a review of worry, prolonged stress-related physiological activation, and health. J Psychosom Res.

[18] Cropley M, Zijlstra FRH, Langan-Fox J, Cooper CL (2011). Work and rumination. Handbook of stress in the occupations.

[19] Åkerstedt T (2006). Psychosocial stress and impaired sleep. Scand J Work Environ Health.

[20] Van Laethem M, Beckers DGJ, Kompier MAJ, Kecklund G, van den Bossche SNJ, Geurts SAE (2015). Bidirectional relations between work-related stress, sleep quality and perseverative cognition. J Psychosom Res.

[21] Pieper S, Brosschot JF, Van der Leeden R, Thayer JF (2007). Cardiac effects of momentary assessed worry episodes and stressful events. Psychosom Med.

[22] Bowling NA, Jex SM, Christiansen ND (2013). The role of personality in occupational stress: a review and future research agenda. Handbook of personality at work.

[23] De Lange AH, Taris TW, Kompier MA, Houtman IL, Bongers PM (2005). Different mechanisms to explain the reversed effects of mental health on work characteristics. Scand J Work Environ Health.

[24] Spector PE, Chen PY, O’Connell BJ (2000). A longitudinal study of relations between job stressors and job strains while controlling for prior negative affectivity and strains. J Appl Psychol..

[25] Meijman TF, Mulder G, Drenth PJD, Thierry H, De Wolff CJ (1998). Psychological aspects of workload. Handbook of work and organizational psychology.

[26] McEwen BS (1998). Stress, adaptation, and disease: allostasis and allostatic load. Ann N Y Acad Sci.

[27] Frese M, Zapf D, Cooper C, Payne R (1988). Methodological issues in the study of work stress: objective vs subjective measurement of work stress and the question of longitudinal studies. Causes, coping and consequences of stress at work.

[28] Zapf D, Dormann C, Frese M (1996). Longitudinal studies in organizational stress research: a review of the literature with reference to methodological issues. J Occup Health Psychol.

[29] Dormann C, Zapf D (2002). Social stressors at work, irritation, and depressive symptoms: accounting for unmeasured third variables in a multi-wave study. J Occup Organ Psychol.

[30] Taris TW, Kompier MAJ (2003). Challenges in longitudinal designs in occupational health psychology. Scand J Work Environ Health.

[31] De Lange AH, Taris TW, Kompier MA, Houtman IL, Bongers PM (2003). “The very best of the millennium”: longitudinal research and the demand-control-(support) model. J Occup Health Psychol.

[32] Åkerstedt T, Nordin M, Alfredsson L, Westerholm P, Kecklund G (2012). Predicting changes in sleep complaints from baseline values and changes in work demands, work control, and work preoccupation—the WOLF-project. Sleep Med.

[33] Nordin M, Åkerstedt T, Nordin S (2013). Psychometric evaluation and normative data for the Karolinska Sleep Questionnaire. Sleep Biol Rhythms.

[34] Chungkham HS, Ingre M, Karasek R, Westerlund H, Theorell T (2013). Factor structure and longitudinal measurement invariance of the demand control support model: an evidence from the Swedish Longitudinal Occupational Survey of Health (SLOSH). PLoS One.

[35] Fransson EI, Nyberg ST, Heikkilä K, Alfredsson L, Bacquer DD, Batty GD (2012). Comparison of alternative versions of the job demand-control scales in 17 European cohort studies: the IPD-work consortium. BMC Public Health.

[36] Theorell T, Perski A, Åkerstedt T, Sigala F, Ahlberg-Hultén G, Svensson J (1988). Changes in job strain in relation to changes in physiological state: a longitudinal study. Scand J Work Environ Health.

[37] Siegrist J, Starke D, Chandola T, Godin I, Marmot M, Niedhammer I (2004). The measurement of effort–reward imbalance at work: European comparisons. Soc Sci Med.

[38] Leineweber C, Wege N, Westerlund H, Theorell T, Wahrendorf M, Siegrist J (2010). How valid is a short measure of effort-reward imbalance at work? A replication study from Sweden. Occup Environ Med.

[39] Lockhart G, MacKinnon DP, Ohlrich V (2011). Mediation analysis in psychosomatic medicine research. Psychosom Med.

[40] Rosseel Y (2012). Lavaan: an R package for structural equation modeling. J Stat Softw.

[41] R Core Team (2015). R: A language and environment for statistical computing.

[42] Arbuckle JL, Marcoulides GA, Schumacker RE (2006). Full information estimation in the presence of incomplete data. Advanced structural equation modeling.

[43] Hu L, Bentler PM (1999). Cutoff criteria for fit indexes in covariance structure analysis: conventional criteria versus new alternatives. Struct Equ Model.

[44] Lichstein KL, Durrence HH, Riedel BW, Taylor DJ, Bush AJ (2004). Epidemiology of sleep: age, gender, and ethnicity.

[45] Prinz PN (2004). Age impairments in sleep, metabolic and immune functions. Exp Gerontol.

[46] MacKinnon DP. Mediation analysis. In: The encyclopedia of clinical psychology. Wiley 2014.

[47] Zhao X, Lynch JG, Chen Q (2010). Reconsidering Baron and Kenny: myths and truths about mediation analysis. J Consum Res.

[48] Hayes AF (2009). Beyond Baron and Kenny: statistical mediation analysis in the new millennium. Commun Monogr.

[49] Hayes AF (2013). An introduction to mediation, moderation, and conditional process analysis: a regression-based approach.

[50] Fairchild AJ, MacKinnon DP, Taborga MP, Taylor AB (2009). R2 effect-size measures for mediation analysis. Behav Res Methods.

[51] Podsakoff PM, Organ DW (1986). Self-reports in organizational research: problems and prospects. J Manag.

[52] Spector PE (2006). Method variance in organizational research: truth or urban legend?. Organ Res Methods.

[53] Van Laethem M, Beckers DGJ, van Hooff MLM, Dijksterhuis A, Geurts SAE (2016). Day-to-day relations between stress and sleep and the mediating role of perseverative cognition. Sleep Med.

[54] Van Hooff MLM, Geurts SAE, Taris TW, Kompier MAJ, Dikkers JSE, Houtman ILD (2005). Disentangling the causal relationships between work-home interference and employee health. Scand J Work Environ Health.

[55] Lallukka T, Rahkonen O, Lahelma E, Arber S (2010). Sleep complaints in middle-aged women and men: the contribution of working conditions and work–family conflicts. J Sleep Res.

[56] Roehrs T, Roth T (2001). Sleep, sleepiness, and alcohol use. Alcohol Res Health.

[57] Sianoja M, Kinnunen U, de Bloom J, Korpela K, Geurts S (2016). Recovery during lunch breaks: testing long-term relations with energy levels at work. Scand J Work Organ Psychol.

[58] Richardson KM, Rothstein HR (2008). Effects of occupational stress management intervention programs: a meta-analysis. J Occup Health Psychol.

[59] Borders A, Earleywine M, Jajodia A (2010). Could mindfulness decrease anger, hostility, and aggression by decreasing rumination?. Aggress Behav.

[60] Jacobs TL, Shaver PR, Epel ES, Zanesco AP, Aichele SR, Bridwell DA (2013). Self-reported mindfulness and cortisol during a Shamatha meditation retreat. Health Psychol.

[61] Jain S, Shapiro SL, Swanick S, Roesch SC, Mills PJ, Bell I (2007). A randomized controlled trial of mindfulness meditation versus relaxation training: effects on distress, positive states of mind, rumination, and distraction. Ann Behav Med.

