# Supplementary material for: Perseverative Cognition as an Explanatory Mechanism in the Relation Between Job Demands and Sleep Quality
Source: Int J Behav Med. 2017 Sep 12;25(2):231–42. doi: 10.1007/s12529-017-9683-y (PMC5852204; doi:10.1007/s12529-017-9683-y)
Supplement: Supplementary file 1 — (DOCX 32 kb) [file 12529_2017_9683_MOESM1_ESM.docx]

**Supplemental material**

Table S1. Means, standard deviations and correlations between research variables.

|  |  | ***M*** | ***SD*** | **1** | **2** | **3** | **4** | **5** | **6** | **7** | **8** | **9** | **10** | **11** | **12** | **13** | **14** | **15** | **16** |
| --- | --- | --- | --- | --- | --- | --- | --- | --- | --- | --- | --- | --- | --- | --- | --- | --- | --- | --- | --- |
| **1** | **Sex**^a^ | 1.59 | 0.49 |  |  |  |  |  |  |  |  |  |  |  |  |  |  |  |  |
| **2** | **Age** | 49.05 | 8.58 | -.01 |  |  |  |  |  |  |  |  |  |  |  |  |  |  |  |
| **3** | **Educational level**  **(1-5)** | 3.28 | 1.36 | .13** | -.14** |  |  |  |  |  |  |  |  |  |  |  |  |  |  |
| **4** | **Work schedule T1^b^** | 0.09 | 0.28 | .09** | .00 | -.15** |  |  |  |  |  |  |  |  |  |  |  |  |  |
| **5** | **Decision authority T1**  **(1-4)** | 3.10 | 0.74 | -.09** | .06** | .14** | -.18** |  |  |  |  |  |  |  |  |  |  |  |  |
| **6** | **Job demands T1**  **(1-4)** | 2.59 | 0.53 | .09** | -.01 | .12** | .04* | -.17** |  |  |  |  |  |  |  |  |  |  |  |
| **7** | **Job demands T2**  **(1-4)** | 2.60 | 0.52 | .08** | -.04* | .08** | .04* | -.13** | .60** |  |  |  |  |  |  |  |  |  |  |
| **8** | **Job demands T3**  **(1-4)** | 2.59 | 0.55 | .09** | -.09** | .07* | .05** | -.14** | .55** | .60** |  |  |  |  |  |  |  |  |  |
| **9** | **Sleep disturbances T1**  **(1-6)** | 2.64 | 1.07 | .13** | .08** | .03 | .04 | -.12** | .29** | .24** | .22** |  |  |  |  |  |  |  |  |
| **10** | **Sleep disturbances T2**  **(1-6)** | 2.62 | 1.05 | .11** | .11** | .01 | .05** | -.08** | .24** | .28** | .24** | .72** |  |  |  |  |  |  |  |
| **11** | **Sleep disturbances T3**  **(1-6)** | 2.64 | 1.05 | .14** | .10** | .01 | .06** | -.11** | .23** | .23** | .27** | .66** | .71** |  |  |  |  |  |  |
| **12** | **Awakening problems T1**  **(1-6)** | 2.67 | 1.08 | .11** | -.17** | .06** | -.01 | -.12** | .25** | .21** | .20** | .46** | .33** | .32** |  |  |  |  |  |
| **13** | **Awakening problems T2**  **(1-6)** | 2.59 | 1.07 | .08** | -.15** | .07** | .00 | -.10** | .21** | .24** | .21** | .35** | .45** | .36** | .68** |  |  |  |  |
| **14** | **Awakening problems T3**  **(1-6)** | 2.57 | 1.06 | .15** | -.14** | .07** | .00 | -.12** | .21** | .22** | .26** | .38** | .39** | .52** | .67** | .72** |  |  |  |
| **15** | **Perseverative cognition T1**  **(1-4)** | 2.23 | 0.80 | .02 | .02 | .16** | -.07** | .00 | .44** | .34** | .29** | .44** | .35** | .32** | .28** | .23** | .22** |  |  |
| **16** | **Perseverative cognition T2**  **(1-4)** | 2.27 | 0.82 | -.01 | -.04* | .14** | -.06** | .04* | .34** | .43** | .33** | .34** | .41** | .32** | .24** | .28** | .23** | .64** |  |
| **17** | **Perseverative cognition T3**  **(1-4)** | 2.24 | 0.82 | .03 | -.04* | .13** | -.05** | .02 | .31** | .34** | .42** | .34** | .35** | .41** | .25** | .25** | .29** | .61** | .67** |

^a^ 1 = male, 2 = female, 52% ♀; b 0 = no shift work, 1= shift work, 8.5% shift work

* = *p* < 0.05, ** = *p* < 0.01 (two-tailed), N = 3017-3080.
