# Supplementary figures and images for: Perseverative Cognition as an Explanatory Mechanism in the Relation Between Job Demands and Sleep Quality
Source: Int J Behav Med. 2017 Sep 12;25(2):231–42. doi: 10.1007/s12529-017-9683-y (PMC5852204; doi:10.1007/s12529-017-9683-y)

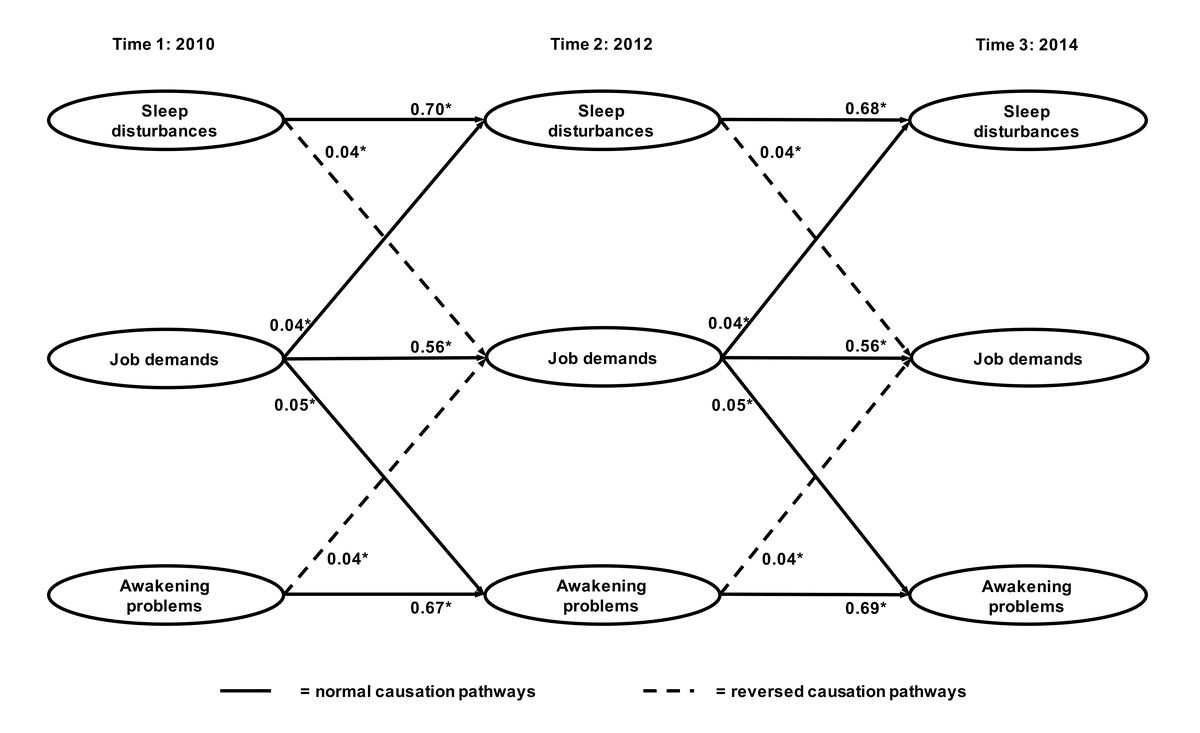

Supplement: Supplementary file 2 — Overview of the normal and reversed pathways and standardized regression coefficients (β). The model is adjusted for age, sex, educational level, work schedule, and decision authority, but for clarity these pathways are not depicted. * = p < 0.05 (GIF 42 kb) [file 12529_2017_9683_MOESM2_ESM.gif]
